# Crystal structure of a 2:1 co-crystal of meloxicam with acetyl­endi­carb­oxy­lic acid

**DOI:** 10.1107/S2056989016018909

**Published:** 2016-11-29

**Authors:** Christian Tantardini, Sergey G. Arkhipov, Ksenya A. Cherkashina, Alexander S. Kil’met’ev, Elena V. Boldyreva

**Affiliations:** a Novosibirsk State University, Pirogova str. 2, Novosibirsk, 630090, Russian Federation; b Institute of Solid State Chemistry and Mechanochemistry SB RAS, Kutateladze str. 18, Novosibirsk, 630128, Russian Federation; c N. N. Vorozhtsov Novosibirsk Institute of Organic Chemistry, Lavrentiev str. 9, Novosibirsk, 630090, Russian Federation

**Keywords:** crystal structure, oxicam, meloxicam, co-crystal, di­carb­oxy­lic acid, acetyl­enedi­carb­oxy­lic acid, hydrogen bonds, drugs

## Abstract

The pharmaceutical 2:1 co-crystal of meloxicam [MXM; systematic name: 4-hy­droxy-2-methyl-*N*-(5-methyl­thia­zol-2-yl)-2*H*-1,2-benzo­thia­zine-3-carboxamide 1,1-dioxide] with acetyl­enedi­carb­oxy­lic acid (ACA; systematic name: but-2-ynedioic acid), crystallizes with one MXM mol­ecule and half an ACA mol­ecule in the asymmetric unit, C_14_H_13_N_3_O_4_S_2_·0.5C_4_H_2_O_4_. The mid-point of the triple bond of ACA is located on an inversion centre. In the crystal, the two stereoisomers of MXM with respect to the N atom of the sulfonamide group are related by the inversion centre. The carbonyl and hy­droxy groups belonging to the MXM mol­ecule are involved in an intra­molecular O—H⋯O hydrogen bond. The structure-forming motif includes two MXM mol­ecules linked *via* an ACA conformer through N—H⋯O and O—H⋯N hydrogen bonds, similar to MXM co-crystals with other di­carb­oxy­lic acids.

## Abstract 2

The crystal structure of a new 2:1 co-crystal of meloxicam and acetyl­endi­carb­oxy­lic acid is reported

## Chemical context   

In recent years, crystal engineering has focused on finding new crystalline forms based on the multi-component crystallization of an active pharmaceutical ingredient (API) with a biologic­ally inactive compound. These complexes are ultimately aimed at being employed in the pharmaceutical industry as tablets, suspensions, powders and any other solid forms for oral administration (Shakhtshneider *et al.*, 2007*a* ▸,*b* ▸; Crowley & Zografi, 2002 ▸; Hancock & Parks, 2000 ▸; Shakhtshneider & Boldyrev, 1993 ▸; Willart & Descamps, 2008 ▸; Shakhtshneider *et al.*, 2011 ▸; Stephenson *et al.*, 2011 ▸). Co-formers are typically chosen from among the di­carb­oxy­lic acids due to their favourable mol­ecular shape and the presence of functional groups capable of forming multiple hydrogen bonds, combined with their affordability and availability. Meloxicam (MXM), 4-hy­droxy-2-methyl-*N*-(5-methyl-2-thia­zol­yl)-2*H*-1,2-benzo­thia­zine-3-carboxamide-1,1-dioxide, belongs to the oxicam family of APIs and is commonly used in the treatment of rheumatoid arthritis (Myz *et al.*, 2012 ▸; Tumanov *et al.*, 2012 ▸; Weyna *et al.* 2012 ▸). MXM is known to co-crystallize with numerous aliphatic and aromatic di­carb­oxy­lic acids under various conditions (temperature, pressure, solvents). In particular, MXM is known to co-crystallize with di­carb­oxy­lic acids of C—C bond order 1 (succinic acid) and 2 (fumaric and maleic acids). The aim of this study was to obtain a co-crystal of MXM with a di­carb­oxy­lic acid of bond order 3: acetyl­enedi­carb­oxy­lic acid (ACA).
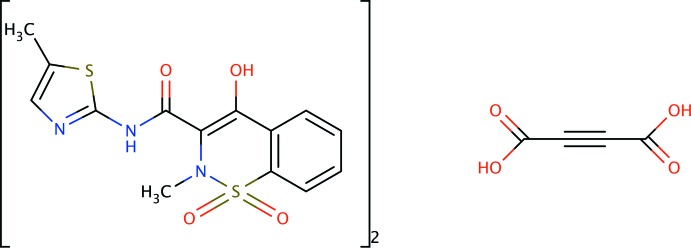


## Structural commentary   

**Figure 1:**
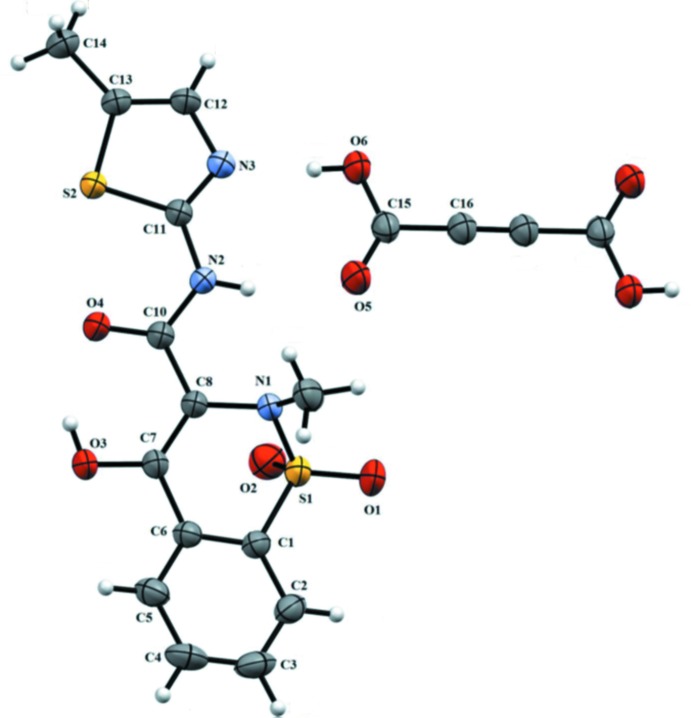
Meloxicam (MXM) and acetyl­enedi­carb­oxy­lic acid (ACA) mol­ecules of the 2:1 co-crystal, showing the atom-numbering scheme. Displacement ellipsoids are drawn at the 50% probability level. Only half of the ACA mol­ecule belongs to the asymmetric unit, as the mol­ecule lies across an inversion centre.

The crystal structure of MXM:ACA 2:1 is triclinic with an asymmetric unit that contains one MXM mol­ecule and half of an ACA mol­ecule. The formula unit is generated by an inversion centre which is located at the midpoint of the triple bond of the ACA mol­ecule (Fig. 1 ▸). The two stereoisomers of MXM, which differ with respect to the nitro­gen atom of the sulfonamide group, are related by an inversion centre in the crystal structure. The dihedral angles between the mean planes of the thia­zole and benzene rings of MXM form an almost planar arrangement in terms of the following torsion angles: S2—C11—N2—H2 = −174.0°, S2—C11—N2—C10 = 6.0 (3)°, H2—N2—C10—O4 = 176.5°, O4—C10—C8—C7 = 10.0 (3)°, C8—C7—O3—H3 = −2.2°. The presence of an intra­molecular O—H⋯O hydrogen bond between the carbonyl and hy­droxy groups belonging to MXM may account for the near planarity and the *trans* position of the N2—H2 group with respect to the carbonyl group C10—O4. The S1/N1/C1/C6/C7/C8 ring is non-planar because of the presence of the sulfonamide group with nitro­gen atom N1 in *sp* ^3^ hybridization, with angles S1—N1—C8 = 112.79 (12)°, S1—N1—C9 = 117.11 (14)° and C9—N1—C8 = 115.41 (17)° (bond-angle sum = 345.3°). The overall conformation of this ring is half-chair with atoms S1 and N1 being the out-of-plane atoms.

## Database survey   

**Figure 2:**
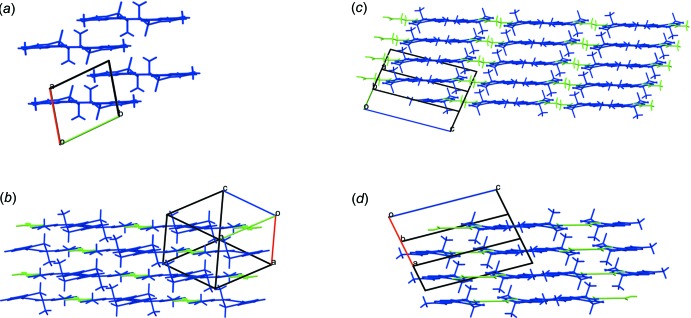
The mol­ecular packing in the crystal structures of (*a*) pure MXM and its co-crystals (*b*) MXM–SUCC, (*c*) MXM–FUM and (*d*) MXM–ACA.

**Figure 3:**
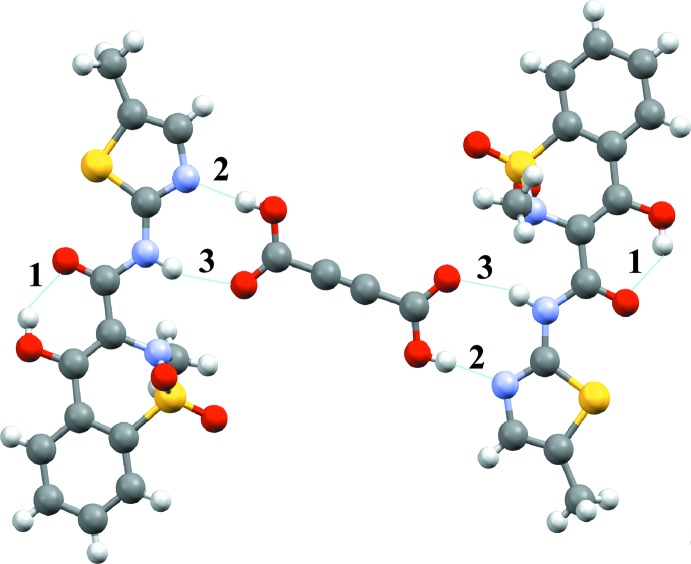
Part of the MXM–ACA 2:1 co-crystal structure showing hydrogen bonds (**1**, **2** and **3**) leading to a trimer. The inter­actions are classified as hydrogen bonds based on the geometric criteria (see text) (Arunan *et al.*, 2011 ▸).

The crystal structures of pure MXM [CCDC ref. code: SEDZOQ (Fabiola *et al.*, 1998 ▸)] and its co-crystals with SUCC (MXM–SUCC) (CCDC ref. code: ENICOUM; Cheney *et al.*, 2010 ▸) and FUM (MXM–FUM) (CCDC ref. code: ENICIO; Cheney *et al.*, 2010 ▸) have the same space group (*P* 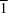). The mol­ecular packing in the title compound is shown in Fig. 2 ▸. It is similar to that in the crystal structures of pure MXM, as well as of MXM–FUM, MXM–SUCC and MXM–ACA (also shown in Fig. 2 ▸). In the co-crystals, some MXM mol­ecules are substituted by the coformer species, maintaining the general packing patterns. The co-crystals MXM–FUM, MXM–SUCC and MXM–ACA have similar structural motifs: two MXM mol­ecules linked by a di­carb­oxy­lic acid mol­ecule (Fig. 3 ▸).

## Supra­molecular features   

In the crystal, the components of the structure are linked by N—H⋯O and O—H⋯N hydrogen bonds between MXM and ACA, in addition to a long O—H⋯O inter­action, forming chains along [011] which incorporates both 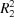
(8) and 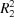
(12) rings. Similar structural motifs have been documented for other MXM co-crystals and in other crystal structures including pure MXM, MXM co-crystals and MXM salts. The structure-forming unit includes two mol­ecules of MXM connected through a di­carb­oxy­lic acid mol­ecule acting as a bridge, similar to what has been reported for other MXM co-crystals (Tumanov *et al.*, 2012 ▸). Intra- and inter­molecular hydrogen bonds are shown in Fig. 3 ▸ and their geometrical parameters are summarized in Table 1 ▸. The centroid-to-centroid distance between symmetry-related benzene and thia­zole rings is 3.7383 (12) Å. These connect the chains into a three-dimensional network.

## Synthesis and crystallization   

MXM was purchased from Sigma Aldrich Co Ltd and acetone from Reaktiv. ACA was synthesized through a two-step process from fumaric acid. Fumaric acid was brominated in boiling water (Rhinesmith, 1938 ▸) and the resulting 2,3-di­bromo­succinic acid was refluxed in potassium hydroxide methano­lic solution. ACA was precipitated by adding a concentrated sulfuric acid solution and dried *in vacuo* (Rhinesmith, 1938 ▸). The purity of ACA and the absence of its monohydrate were checked by comparing its experimental powder X-ray diffraction powder (XRPD) pattern with the calculated XRPD patterns of ACA and ACA monohydrate (see S1 in Supporting information). Two polycrystalline samples were obtained by dry and slurry (with acetone) grinding of 1:2 molar mixture of reactants (0.035g, 0.1mmol MXM; 0.023g, 0.2mmol ACA). The 2:1 ratio would correspond to the target stoichiometry and is usually used for obtaining other MXM co-crystals with aliphatic di­carb­oxy­lic acids (Myz *et al.*, 2012 ▸; Tumanov *et al.*, 2012 ▸; Weyna *et al.* 2012 ▸). However, to obtain MXM–ACA 2:1 co-crystals we used a 1:2 MXM:ACA ratio because ACA is highly hygroscopic and converts to its monohydrate form on grinding, not participating then in the co-crystallization. Acetone was used for slurry grinding because it completely dissolves the two starting components (Myz *et al.*, 2012 ▸; Tumanov *et al.*, 2012 ▸; Weyna *et al.* 2012 ▸). All powder samples were characterized by XRPD using a Stoe Stadi-MP diffractometer with Cu *K*α_1_ radiation (λ = 1.54060 Å) at operating potential of 40 kV and electric current of 40 mA, and a Mythen 1K detector. All data were processed using *WinXPOW* (Stoe & Cie, 1999 ▸). Powder diffraction patterns for the samples obtained by grinding and slurry grinding were similar, confirming the possibility to obtain the same product both in the presence and in the absence of a specially added solvent (see S2 in Supporting information); the XRPD patterns of the co-crystal sample were compared with the patterns of the starting reactants, MXM and ACA (see S3 in Supporting information) to prove that a new phase (or a mixture of new phases) had been formed. The ground powder samples were subsequently dissolved in acetone and single crystals were obtained by slow evaporation. Selected crystals were investigated using single-crystal X-ray diffraction.

## Refinement   

Crystal data, data collection and structure refinement details are summarized in Table 2 ▸.

All H atoms were initially located in a difference Fourier map. The positions of all H atoms were subsequently optimized geometrically and refined using a riding model, with the following assumptions and restraints: N—H = 0.86 Å and *U*_iso_(H)=1.2*U*_eq_(N) for —N(H)– group, C—H = 0.93 Å and *U*_iso_(H) = 1.2*U*eq(C) for all C—H groups, O—H = 0.82 Å and *U*_iso_(H) = 1.5*U*_eq_(O) for all OH groups, C—H = 0.96 Å and *U*_iso_(H) = 1.5*U*_eq_(C) for CH_3_ groups.

For single crystals of MXM:ACA (2:1), two data sets were collected. The first dataset was obtained from a crystal containing four domains, and the second from a single crystal. Unfortunately, the single crystal was very small and at *d*_hkl_ ≥ 0.80 Å, *R*_int_ was 10.2% and *F* ^2^/σ(*F* ^2^) was 3.6. This was significantly worse than the data from the crystal that contained four domains [for the largest domain at *d*_hkl_ ≥ 0.80 Å, *R*_int_ was 2.50% and *F* ^2^/σ(*F* ^2^) was 28.3]. Data obtained from the crystal that contained four domains were processed in three different ways: (1) taking into account the reflections from the largest domain only (one orientation matrix and 74.3% of all reflections); (2) processing the diffraction data as from multiple crystals (four different orientation matrices) using the hklf5-file; (3) processing the diffraction data as from multiple crystals (4 different orientation matrixes) using the. hklf4-file from the largest domain (74.3% of all reflections). The first and the third processing methods gave approximately the same results, while the first methodology yielded the best results: *R*_int_ = 0.025. This method was therefore chosen for the final structure solution and refinement.

The powder diffraction patterns calculated based on the X-ray single crystal diffraction data were compared with the experimental powder diffraction pattern measured for the sample obtained on grinding, to show that the latter contained a mixture of the MXM:ACA 2:1 co-crystal with some other phases, different from ACA, MXM, or ACA hydrate (see S4 in Supporting information).

## Supplementary Material

Crystal structure: contains datablock(s) I. DOI: 10.1107/S2056989016018909/lh5829sup1.cif

Structure factors: contains datablock(s) I. DOI: 10.1107/S2056989016018909/lh5829Isup2.hkl

S1. X-ray powder diffraction pattern calculated for ACA from single-crystal diffraction data (1) (ACEDAC01) and the experimental XRPD patterns of ACA (2) and its monohydrate (3). S2. XRPD patterns of (1:2) MXM and ACA mixtures after grinding (1) and slurry grinding (acetone) (2). S3. XRPD patterns of ACA (1), MXM (2) and a sample obtained through slurry grinding (acetone) of a of (1:2) MXM and ACA mixture (3). S4. An XRPD pattern calculated for MXM:ACA (2:1) co-crystal based on single-crystal X-ray diffraction data (1) and an experimental XRPD pattern measured for a MXM and ACA (1:2) powder mixture after slurry grinding (acetone). The red arrows indicate the presence of the peaks not belonging to the MXM:ACA 2:1 co-crystal, corresponding to some other phases.. DOI: 10.1107/S2056989016018909/lh5829sup3.pdf

Supporting information file. DOI: 10.1107/S2056989016018909/lh5829Isup4.cml

CCDC reference: 1506179

Additional supporting information: crystallographic information; 3D view; checkCIF report

## Figures and Tables

**Table 1:** Geometrical parameters (Å, °) for the O—H⋯O (**1**), O—H⋯N (**2**) and N—H⋯O (**3**) inter­actions in the MXM:ACA 2:1 co-crystal (see also Fig. 3 ▸)

D—H⋯A	D—H	H⋯A	D⋯A	D—H⋯A
O3—H3⋯O4 (**1**)	0.82	1.91	2.622 (2)	145
O6—H6⋯N3 (**2**)	0.82	1.80	2.615 (3)	174
N2—H2⋯O5 (**3**)	0.86	2.09	2.922 (3)	164
O3—H3⋯O4^i^	0.82	2.51	2.944 (2)	114

Symmetry code: (i) −*x* + 1, −*y* + 1, −*z*.

**Table 2:** Experimental details

Crystal data	
Chemical formula	C_14_H_13_N_3_O_4_S_2_·0.5C_4_H_2_O_4_
*M*_r_	408.42
Crystal system, space group	Triclinic, *P* 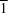
Temperature (K)	293
*a*, *b*, *c* (Å)	7.3861 (3), 8.5629 (3), 15.1619 (6)
α, β, γ (°)	75.839 (3), 79.196 (3), 70.100 (3)
*V* (Å^3^)	868.55 (6)
*Z*	2
Radiation type	Mo *K*α
μ (mm^−1^)	0.35
Crystal size (mm)	0.4 × 0.25 × 0.1

Data collection	
Diffractometer	Agilent Xcalibur (Ruby, Gemini ultra)
Absorption correction	Multi-scan (*CrysAlis PRO*; Agilent, 2013 ▸)
*T*_min_, *T*_max_	0.982, 1.000
No. of measured, independent and observed [*I* > 2σ(*I*)] reflections	10893, 3564, 2940
*R*_int_	0.025
(sin θ/λ)_max_ (Å^−1^)	0.625

Refinement	
*R*[*F* ^2^ > 2σ(*F* ^2^)], *wR*(*F* ^2^), *S*	0.036, 0.098, 1.05
No. of reflections	3564
No. of parameters	248
H-atom treatment	H-atom parameters constrained
Δρ_max_, Δρ_min_ (e Å^−3^)	0.35, −0.30

Computer programs: *CrysAlis PRO* (Agilent, 2013 ▸), *SHELXS97* (Sheldrick, 2008 ▸), *SHELXL2014* (Sheldrick, 2015 ▸), *OLEX2* (Dolomanov *et al.*, 2009 ▸), *Mercury* (Macrae *et al.*, 2008 ▸ and *publCIF* (Westrip, 2010 ▸).

## supplementary crystallographic information

### Crystal data

**Table:**

C_14_H_13_N_3_O_4_S_2_·0.5C_4_H_2_O_4_	*Z* = 2
*M**_r_* = 408.42	*F*(000) = 422
Triclinic, *P*1	*D*_x_ = 1.562 Mg m^−^^3^
*a* = 7.3861 (3) Å	Mo *K*α radiation, λ = 0.71073 Å
*b* = 8.5629 (3) Å	Cell parameters from 4820 reflections
*c* = 15.1619 (6) Å	θ = 2.6–28.0°
α = 75.839 (3)°	µ = 0.35 mm^−^^1^
β = 79.196 (3)°	*T* = 293 K
γ = 70.100 (3)°	Prism, clear light colourless
*V* = 868.55 (6) Å^3^	0.4 × 0.25 × 0.1 mm

### Data collection

**Table:**

Agilent Xcalibur (Ruby, Gemini ultra) diffractometer	3564 independent reflections
Radiation source: Enhance (Mo) X-ray Source	2940 reflections with *I* > 2σ(*I*)
Graphite monochromator	*R*_int_ = 0.025
Detector resolution: 10.3457 pixels mm^-1^	θ_max_ = 26.4°, θ_min_ = 2.6°
ω scans	*h* = −9→9
Absorption correction: multi-scan (CrysAlis PRO; Agilent, 2013)	*k* = −10→10
*T*_min_ = 0.982, *T*_max_ = 1.000	*l* = −18→18
10893 measured reflections	

### Refinement

**Table:**

Refinement on *F*^2^	Primary atom site location: structure-invariant direct methods
Least-squares matrix: full	Hydrogen site location: inferred from neighbouring sites
*R*[*F*^2^ > 2σ(*F*^2^)] = 0.036	H-atom parameters constrained
*wR*(*F*^2^) = 0.098	*w* = 1/[σ^2^(*F*_o_^2^) + (0.0463*P*)^2^ + 0.2383*P*] where *P* = (*F*_o_^2^ + 2*F*_c_^2^)/3
*S* = 1.05	(Δ/σ)_max_ < 0.001
3564 reflections	Δρ_max_ = 0.35 e Å^−^^3^
248 parameters	Δρ_min_ = −0.30 e Å^−^^3^
0 restraints	

### Special details

**Table:**

Experimental. Suitable-quality crystals were selected using polarised light under the microscope and mounted by means of MiTiGenMicroGrippers using MiTiGen LV Cryo Oil (LVCO-1) onto an Agilent Xcalibur (Ruby, Gemini Ultra) diffractometer.
Geometry. All esds (except the esd in the dihedral angle between two l.s. planes) are estimated using the full covariance matrix. The cell esds are taken into account individually in the estimation of esds in distances, angles and torsion angles; correlations between esds in cell parameters are only used when they are defined by crystal symmetry. An approximate (isotropic) treatment of cell esds is used for estimating esds involving l.s. planes.

### Fractional atomic coordinates and isotropic or equivalent isotropic displacement parameters (Å^2^)

**Table:**

	*x*	*y*	*z*	*U*_iso_*/*U*_eq_	
S2	0.26125 (7)	0.95843 (6)	0.03447 (3)	0.03606 (14)	
S1	0.83244 (8)	0.38866 (6)	0.33477 (3)	0.04072 (15)	
O4	0.4415 (2)	0.61781 (17)	0.08344 (9)	0.0447 (4)	
O3	0.6628 (2)	0.30130 (18)	0.10423 (10)	0.0463 (4)	
H3	0.591499	0.389131	0.077100	0.069*	
O6	0.3828 (2)	1.10848 (17)	0.32189 (10)	0.0439 (4)	
H6	0.367153	1.088861	0.274023	0.066*	
N2	0.4321 (2)	0.76888 (19)	0.18889 (11)	0.0350 (4)	
H2	0.457747	0.764077	0.242714	0.042*	
N3	0.3138 (2)	1.06452 (19)	0.16821 (11)	0.0353 (4)	
C10	0.4825 (3)	0.6209 (2)	0.15831 (13)	0.0336 (4)	
C11	0.3423 (3)	0.9263 (2)	0.13860 (12)	0.0308 (4)	
C13	0.1832 (3)	1.1746 (2)	0.03060 (13)	0.0339 (4)	
N1	0.6110 (2)	0.48326 (19)	0.30707 (10)	0.0375 (4)	
O2	0.9585 (2)	0.46318 (19)	0.26770 (11)	0.0517 (4)	
C12	0.2232 (3)	1.2058 (2)	0.10662 (13)	0.0372 (4)	
H12	0.191841	1.315071	0.116962	0.045*	
C8	0.5911 (3)	0.4690 (2)	0.21752 (12)	0.0334 (4)	
C6	0.7971 (3)	0.1699 (2)	0.24281 (13)	0.0320 (4)	
O5	0.4837 (2)	0.82648 (18)	0.36346 (10)	0.0529 (4)	
C7	0.6775 (3)	0.3215 (2)	0.18691 (13)	0.0331 (4)	
C1	0.8718 (3)	0.1834 (2)	0.31771 (13)	0.0337 (4)	
C15	0.4507 (3)	0.9670 (2)	0.37751 (13)	0.0355 (4)	
C16	0.4862 (3)	0.9913 (2)	0.46450 (13)	0.0379 (4)	
O1	0.8318 (3)	0.3844 (2)	0.42935 (10)	0.0621 (5)	
C2	0.9717 (3)	0.0418 (3)	0.37611 (14)	0.0423 (5)	
H2A	1.018860	0.053179	0.426149	0.051*	
C5	0.8338 (3)	0.0088 (2)	0.22541 (16)	0.0419 (5)	
H5	0.791005	−0.003525	0.174357	0.050*	
C14	0.0890 (3)	1.2967 (3)	−0.04937 (14)	0.0443 (5)	
H14A	0.153415	1.258732	−0.104979	0.066*	
H14B	0.098095	1.406802	−0.051516	0.066*	
H14C	−0.044963	1.302810	−0.042813	0.066*	
C3	1.0008 (3)	−0.1169 (3)	0.35939 (16)	0.0506 (6)	
H3A	1.065483	−0.213032	0.399023	0.061*	
C4	0.9342 (3)	−0.1333 (3)	0.28424 (17)	0.0492 (6)	
H4	0.956697	−0.240531	0.272748	0.059*	
C9	0.4499 (3)	0.4608 (3)	0.37857 (15)	0.0503 (6)	
H9A	0.456712	0.343501	0.394250	0.075*	
H9B	0.328377	0.527269	0.355785	0.075*	
H9C	0.460436	0.497153	0.431969	0.075*	

### Atomic displacement parameters (Å^2^)

**Table:**

	*U*^11^	*U*^22^	*U*^33^	*U*^12^	*U*^13^	*U*^23^
S2	0.0495 (3)	0.0272 (3)	0.0303 (3)	−0.0048 (2)	−0.0144 (2)	−0.00641 (19)
S1	0.0582 (3)	0.0324 (3)	0.0323 (3)	−0.0072 (2)	−0.0181 (2)	−0.0080 (2)
O4	0.0633 (9)	0.0325 (7)	0.0356 (8)	−0.0010 (6)	−0.0228 (7)	−0.0089 (6)
O3	0.0660 (10)	0.0363 (8)	0.0350 (8)	0.0000 (7)	−0.0215 (7)	−0.0148 (6)
O6	0.0624 (9)	0.0372 (8)	0.0364 (8)	−0.0121 (7)	−0.0180 (7)	−0.0106 (6)
N2	0.0472 (9)	0.0265 (8)	0.0282 (8)	−0.0024 (7)	−0.0135 (7)	−0.0055 (6)
N3	0.0457 (9)	0.0275 (8)	0.0322 (9)	−0.0066 (7)	−0.0112 (7)	−0.0066 (7)
C10	0.0408 (10)	0.0284 (10)	0.0299 (10)	−0.0046 (8)	−0.0096 (8)	−0.0071 (8)
C11	0.0357 (10)	0.0264 (9)	0.0285 (10)	−0.0046 (7)	−0.0073 (8)	−0.0067 (7)
C13	0.0396 (10)	0.0265 (9)	0.0321 (10)	−0.0052 (8)	−0.0080 (8)	−0.0036 (8)
N1	0.0532 (10)	0.0279 (8)	0.0258 (8)	−0.0002 (7)	−0.0103 (7)	−0.0086 (6)
O2	0.0613 (10)	0.0429 (9)	0.0583 (10)	−0.0217 (7)	−0.0158 (8)	−0.0085 (7)
C12	0.0489 (11)	0.0239 (9)	0.0365 (11)	−0.0056 (8)	−0.0104 (9)	−0.0054 (8)
C8	0.0432 (10)	0.0270 (9)	0.0275 (10)	−0.0040 (8)	−0.0097 (8)	−0.0065 (7)
C6	0.0355 (10)	0.0252 (9)	0.0323 (10)	−0.0057 (7)	−0.0031 (8)	−0.0060 (7)
O5	0.0811 (11)	0.0372 (9)	0.0457 (9)	−0.0123 (8)	−0.0245 (8)	−0.0130 (7)
C7	0.0420 (10)	0.0295 (10)	0.0289 (10)	−0.0082 (8)	−0.0095 (8)	−0.0075 (7)
C1	0.0382 (10)	0.0279 (10)	0.0296 (10)	−0.0049 (8)	−0.0042 (8)	−0.0035 (7)
C15	0.0373 (10)	0.0382 (11)	0.0335 (11)	−0.0104 (8)	−0.0060 (8)	−0.0117 (8)
C16	0.0454 (11)	0.0360 (11)	0.0353 (10)	−0.0126 (9)	−0.0092 (9)	−0.0085 (9)
O1	0.0908 (13)	0.0541 (10)	0.0382 (9)	−0.0004 (9)	−0.0318 (8)	−0.0160 (7)
C2	0.0427 (11)	0.0398 (12)	0.0330 (11)	−0.0022 (9)	−0.0064 (9)	0.0002 (9)
C5	0.0437 (11)	0.0300 (10)	0.0509 (13)	−0.0066 (8)	−0.0056 (9)	−0.0131 (9)
C14	0.0556 (13)	0.0324 (11)	0.0392 (12)	−0.0047 (9)	−0.0152 (10)	−0.0020 (9)
C3	0.0471 (12)	0.0329 (11)	0.0521 (14)	0.0017 (9)	−0.0045 (10)	0.0064 (10)
C4	0.0489 (12)	0.0241 (10)	0.0668 (16)	−0.0041 (9)	−0.0028 (11)	−0.0078 (10)
C9	0.0668 (15)	0.0407 (12)	0.0338 (12)	−0.0061 (10)	0.0005 (10)	−0.0099 (9)

### Geometric parameters (Å, º)

**Table:**

S2—C11	1.7196 (18)	C8—C7	1.359 (2)
S2—C13	1.7306 (18)	C6—C7	1.464 (3)
S1—N1	1.6422 (17)	C6—C1	1.397 (3)
S1—O2	1.4284 (16)	C6—C5	1.394 (3)
S1—C1	1.7567 (19)	O5—C15	1.208 (2)
S1—O1	1.4246 (15)	C1—C2	1.382 (3)
O4—C10	1.237 (2)	C15—C16	1.467 (3)
O3—H3	0.8200	C16—C16^i^	1.185 (4)
O3—C7	1.335 (2)	C2—H2A	0.9300
O6—H6	0.8200	C2—C3	1.381 (3)
O6—C15	1.294 (2)	C5—H5	0.9300
N2—H2	0.8600	C5—C4	1.389 (3)
N2—C10	1.364 (2)	C14—H14A	0.9600
N2—C11	1.384 (2)	C14—H14B	0.9600
N3—C11	1.304 (2)	C14—H14C	0.9600
N3—C12	1.383 (2)	C3—H3A	0.9300
C10—C8	1.459 (2)	C3—C4	1.376 (3)
C13—C12	1.349 (3)	C4—H4	0.9300
C13—C14	1.500 (3)	C9—H9A	0.9600
N1—C8	1.431 (2)	C9—H9B	0.9600
N1—C9	1.482 (3)	C9—H9C	0.9600
C12—H12	0.9300		
			
C11—S2—C13	89.50 (9)	O3—C7—C8	123.70 (17)
N1—S1—C1	100.92 (9)	O3—C7—C6	114.19 (16)
O2—S1—N1	107.52 (9)	C8—C7—C6	122.11 (17)
O2—S1—C1	108.23 (9)	C6—C1—S1	117.03 (14)
O1—S1—N1	108.86 (9)	C2—C1—S1	121.39 (16)
O1—S1—O2	119.90 (11)	C2—C1—C6	121.57 (18)
O1—S1—C1	109.73 (10)	O6—C15—C16	112.85 (17)
C7—O3—H3	109.5	O5—C15—O6	126.38 (18)
C15—O6—H6	109.5	O5—C15—C16	120.77 (18)
C10—N2—H2	118.0	C16^i^—C16—C15	178.9 (3)
C10—N2—C11	124.02 (16)	C1—C2—H2A	120.4
C11—N2—H2	118.0	C3—C2—C1	119.3 (2)
C11—N3—C12	110.79 (16)	C3—C2—H2A	120.4
O4—C10—N2	121.31 (16)	C6—C5—H5	120.0
O4—C10—C8	122.38 (16)	C4—C5—C6	120.1 (2)
N2—C10—C8	116.29 (16)	C4—C5—H5	120.0
N2—C11—S2	124.41 (13)	C13—C14—H14A	109.5
N3—C11—S2	114.56 (13)	C13—C14—H14B	109.5
N3—C11—N2	121.03 (16)	C13—C14—H14C	109.5
C12—C13—S2	109.53 (14)	H14A—C14—H14B	109.5
C12—C13—C14	129.41 (17)	H14A—C14—H14C	109.5
C14—C13—S2	121.06 (14)	H14B—C14—H14C	109.5
C8—N1—S1	112.79 (12)	C2—C3—H3A	119.9
C8—N1—C9	115.41 (17)	C4—C3—C2	120.20 (19)
C9—N1—S1	117.11 (14)	C4—C3—H3A	119.9
N3—C12—H12	122.2	C5—C4—H4	119.7
C13—C12—N3	115.61 (17)	C3—C4—C5	120.6 (2)
C13—C12—H12	122.2	C3—C4—H4	119.7
N1—C8—C10	117.67 (15)	N1—C9—H9A	109.5
C7—C8—C10	120.92 (16)	N1—C9—H9B	109.5
C7—C8—N1	121.34 (16)	N1—C9—H9C	109.5
C1—C6—C7	120.26 (16)	H9A—C9—H9B	109.5
C5—C6—C7	121.56 (18)	H9A—C9—H9C	109.5
C5—C6—C1	118.14 (17)	H9B—C9—H9C	109.5
			
S2—C13—C12—N3	0.0 (2)	O2—S1—C1—C2	105.81 (18)
S1—N1—C8—C10	−135.40 (16)	C12—N3—C11—S2	−0.1 (2)
S1—N1—C8—C7	41.7 (2)	C12—N3—C11—N2	179.66 (17)
S1—C1—C2—C3	−179.35 (15)	C6—C1—C2—C3	0.9 (3)
O4—C10—C8—N1	−172.87 (18)	C6—C5—C4—C3	−0.9 (3)
O4—C10—C8—C7	10.0 (3)	C7—C6—C1—S1	−5.1 (2)
N2—C10—C8—N1	8.8 (3)	C7—C6—C1—C2	174.63 (18)
N2—C10—C8—C7	−168.30 (18)	C7—C6—C5—C4	−174.64 (18)
C10—N2—C11—S2	6.0 (3)	C1—S1—N1—C8	−54.93 (15)
C10—N2—C11—N3	−173.71 (17)	C1—S1—N1—C9	82.64 (15)
C10—C8—C7—O3	−3.3 (3)	C1—C6—C7—O3	161.27 (17)
C10—C8—C7—C6	176.22 (18)	C1—C6—C7—C8	−18.3 (3)
C11—S2—C13—C12	−0.05 (15)	C1—C6—C5—C4	3.0 (3)
C11—S2—C13—C14	179.40 (17)	C1—C2—C3—C4	1.3 (3)
C11—N2—C10—O4	−3.5 (3)	O1—S1—N1—C8	−170.34 (14)
C11—N2—C10—C8	174.84 (17)	O1—S1—N1—C9	−32.77 (17)
C11—N3—C12—C13	0.0 (3)	O1—S1—C1—C6	153.05 (15)
C13—S2—C11—N2	−179.64 (17)	O1—S1—C1—C2	−26.7 (2)
C13—S2—C11—N3	0.07 (15)	C2—C3—C4—C5	−1.3 (3)
N1—S1—C1—C6	38.29 (16)	C5—C6—C7—O3	−21.1 (3)
N1—S1—C1—C2	−141.46 (17)	C5—C6—C7—C8	159.28 (19)
N1—C8—C7—O3	179.69 (18)	C5—C6—C1—S1	177.20 (15)
N1—C8—C7—C6	−0.8 (3)	C5—C6—C1—C2	−3.0 (3)
O2—S1—N1—C8	58.34 (15)	C14—C13—C12—N3	−179.36 (19)
O2—S1—N1—C9	−164.09 (14)	C9—N1—C8—C10	86.3 (2)
O2—S1—C1—C6	−74.44 (16)	C9—N1—C8—C7	−96.7 (2)

Symmetry code: (i) −*x*+1, −*y*+2, −*z*+1.

### Hydrogen-bond geometry (Å, º)

**Table:**

*D*—H···*A*	*D*—H	H···*A*	*D*···*A*	*D*—H···*A*
O3—H3···O4	0.82	1.91	2.6221 (18)	145
O6—H6···N3	0.82	1.80	2.615 (2)	174
N2—H2···O5	0.86	2.09	2.922 (2)	164
O3—H3···O4^ii^	0.82	2.51	2.944 (2)	114

Symmetry code: (ii) −*x*+1, −*y*+1, −*z*.
