# Supplementary material for: Crystal structure of a 2:1 co-crystal of meloxicam with acetyl­endi­carb­oxy­lic acid
Source: Acta Crystallogr E Crystallogr Commun. 2016 Nov 29;72(Pt 12):1856–9. doi: 10.1107/S2056989016018909 (PMC5137627; doi:10.1107/S2056989016018909)

Supporting information

S1. X-ray powder diffraction pattern calculated for ACA from single-crystal diffraction data (1) (ACEDAC01) and the experimental XRPD patterns of ACA (2) and its monohydrate (3)

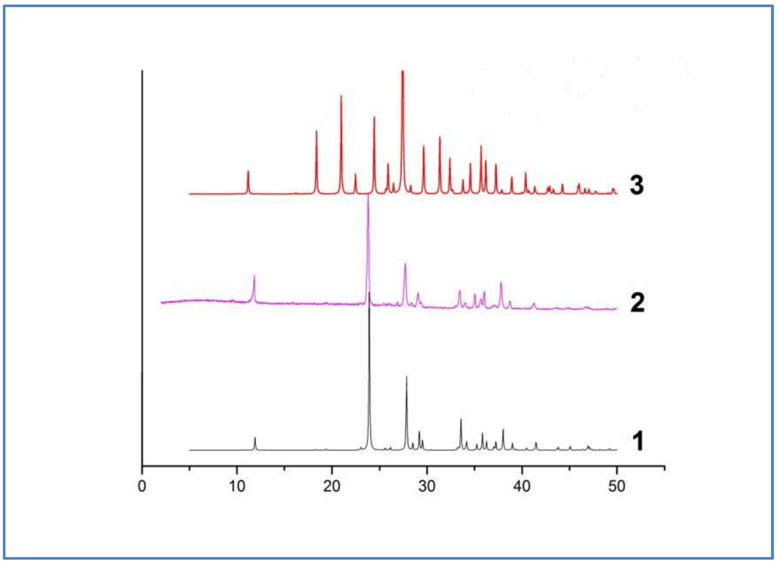

S2. XRPD patterns of (1:2) MXM & ACA mixtures after grinding (1) and slurry grinding (acetone) (2).

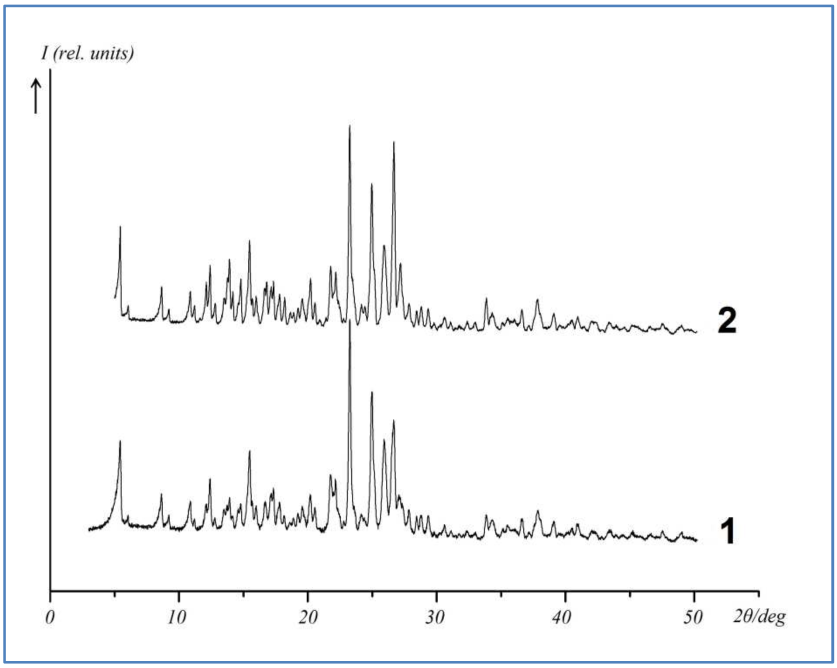

IMPORTANT: this document contains embedded data - to preserve data integrity, please ensure where possible that the IUCr Word tools (available from http://journals.iucr.org/services/docxtemplate/) are installed when editing this document.

S3. XRPD patterns of ACA (1), MXM (2) and a sample obtained through slurry grinding (acetone) of a of (1:2) MXM & ACA mixture (3).

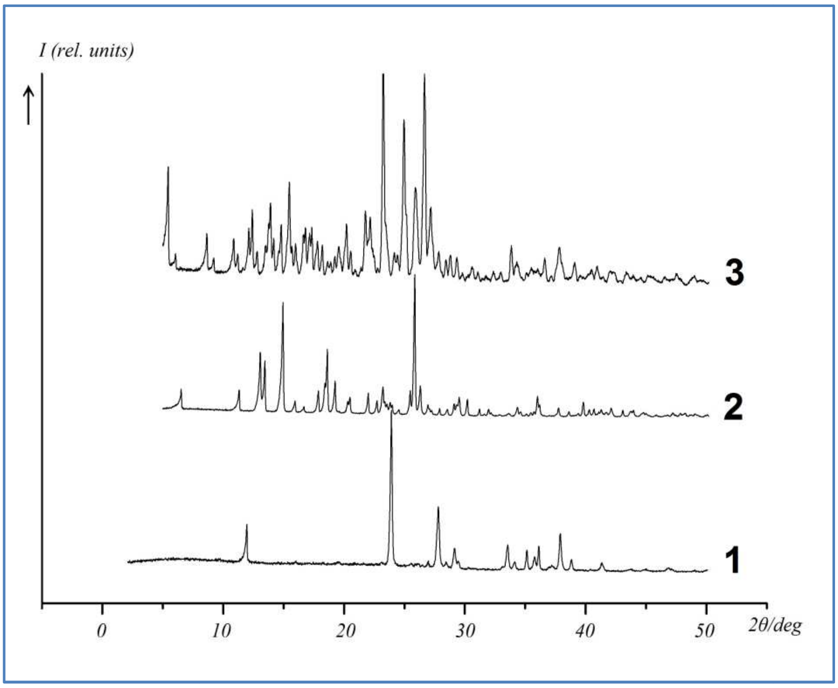

S4. An XRPD pattern calculated for MXM:ACA (2:1) cocrystal based on single-crystal X-ray diffraction data (1) and an experimental XRPD pattern measured for a MXM & ACA (1:2) powder mixture after slurry grinding (acetone). The red arrows indicate the presence of the peaks not belonging to the MXM:ACA 2:1 cocrystal, corresponding to some other phases.

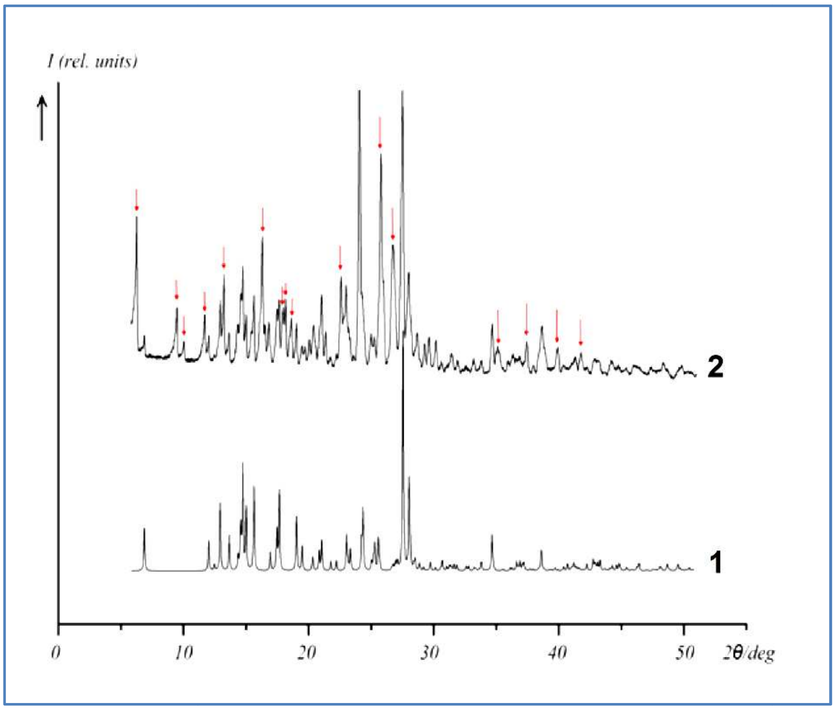

Supplement: Supplementary file 3 [file e-72-01856-sup3.pdf]
